# Asymmetric adhesive SIS-based wound dressings for therapeutically targeting wound repair

**DOI:** 10.1186/s12951-024-02294-x

**Published:** 2024-01-19

**Authors:** Wende Yao, Zelong Song, Xiaodong Ma, Yiqian Huang, Xueying Zhang, Yunhuan Li, Pengfei Wei, Julei Zhang, Chenlu Xiong, Sihan Yang, Yujian Xu, Wei Jing, Bo Zhao, Xuesong Zhang, Yan Han

**Affiliations:** 1 School of Medicine, Nankai University, Tianjin, 300071 China; 2 Department of Plastic and Reconstructive Surgery, The First Medical Centre, Chinese PLA General Hospital, Beijing, 100853 China; 3 Department of Orthopaedics, The Fourth Medical Centre, Chinese PLA General Hospital, Beijing, 100048 China; 4 Department of Neurosurgery, The First Medical Centre, Chinese PLA General Hospital, Beijing, 100048 China; 5 Beijing Biosis Healing Biological Technology Co., Ltd, Beijing, 102600 China; 6 Department of Burn and Plastic Surgery, The 980st Hospital of the PLA Joint Logistics Support Force, Hebei, China

**Keywords:** Wound dressing, Asymmetric composite, SIS, Vascularization, Tissue regeneration

## Abstract

Severe tissue injuries pose a significant risk to human health. Conventional wound dressings fall short in achieving effective tissue regeneration, resulting in suboptimal postoperative healing outcomes. In this study, an asymmetric adhesive wound dressing (marked as SIS/PAA/LAP) was developed, originating from acrylate acid (AA) solution with laponite (LAP) nanoparticles polymerization and photo-crosslinked on the decellularized extracellular matrix small intestinal submucosa (SIS) patch. Extensive studies demonstrated that the SIS/PAA/LAP exhibited higher tissue adhesion strength (~ 33 kPa) and burst strength (~ 22 kPa) compared to conventional wound dressings like Tegaderm and tissue adhesive products. Importantly, it maintained favorable cell viability and demonstrated robust angiogenic capacity. In animal models of full-thickness skin injuries in rats and skin injuries in Bama miniature pigs, the SIS/PAA/LAP could be precisely applied to wound sites. By accelerating the formation of tissue vascularization, it displayed superior tissue repair outcomes. This asymmetrically adhesive SIS-based patch would hold promising applications in the field of wound dressings.

**Supplementary Information:**

The online version contains supplementary material available at 10.1186/s12951-024-02294-x.

## Introduction

Human skin serves multiple vital functions, such as protecting the body from bacterial invasion, regulating body temperature, preserving moisture, and acting as the body’s largest interface with external environment [1]. When the skin suffers severe trauma, the wound healing process does not recover naturally, orderly, and perfectly. Various adverse factors can lead to irregular and not ideal wound healing. In such cases, despite the use of surgical sutures, wound dressings, and other instruments to perform surgical closure and isolate the wound from microbial contamination, remains the gold standard for treating skin injuries. However, this approach demands significant clinical costs and patient burdens [2, 3]. Decellularized extracellular matrix materials, as naturally-derived cell scaffolding carriers, exhibit excellent biocompatibility and biodegradability, which have been widely used in the treatment and care of various wounds and burns, such as Biodesign™ (COOK Corp., USA) and Gentrix® (Integra Corp., USA) [4, 5]. These extracellular matrix materials retain a suitable spatial structure for cell growth, and offer a rough, porous fibrous morphology that provides porosity and a permeable environment, contributing to the acceleration of wound healing [6–8].

While extracellular matrix materials have made significant strides in clinical and product translation, the majority of these materials primarily act as “passive” carriers for facilitating tissue regeneration by creating a favorable environment for cell growth [9], that is, in most cases, extracellular matrix materials function as physical barriers for wound healing. In recent years, researchers have been incorporating active components into extracellular matrix materials, such as drugs, functional peptides, exosomes, etc., to enhances these scaffolding materials with exceptional antibacterial activity, angiogenic capacities, and the ability to recruit cells, thereby achieving more effective wound healing promotion [10–16]. Among these active components, laponite (LAP) nanomaterial, as a common natural clay, which is rich in active elements such as magnesium (Mg) and silicon (Si), is considered an ideal candidate for accelerating tissue regeneration [17, 18]. Due to the presence of inherent electrostatic interactions on its surface, it can self-assemble into a hydrogel at certain concentrations. As a result, it has widespread application in tissue engineering fields, such as for skin, cardiovascular, and bone tissue engineering [19].

**Scheme 1:**
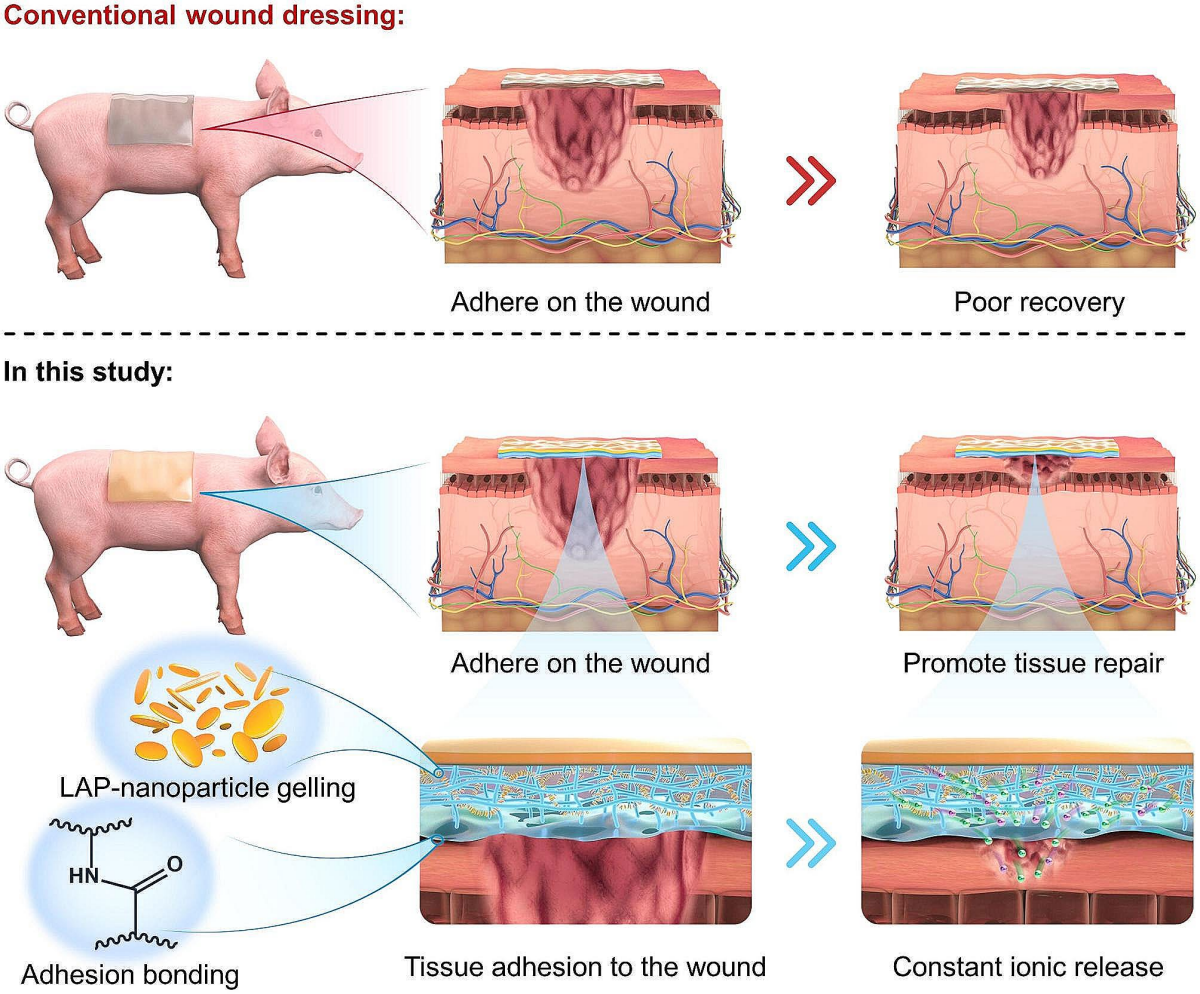
Conventional wound dressings resulted in poor tissue recovery due to easy tissue failure and lack of regenerative induction. In this study, an asymmetric adhesive SIS-based wound dressing containing LAP nanoparticles (with Mg and Si) was prepared and applied to the wound, and resulted in tissue regeneration due to robust adhesion to the injury and bioactive ion release

In this study, a mixture of acrylate acid (AA) and LAP solutions was applied coating onto decellularized extracellular matrix material from porcine small intestinal submucosa (SIS). Under ultraviolet light exposure, it led to the preparation of asymmetrically adhesive SIS-based wound dressing. The composite wound dressing, thanks to the dual-network structure consisting of the physical gel of LAP and the chemical gel of polyacrylic acid (PAA), improved the mechanical properties and tissue adhesion of the material. The adhesion results outperformed commercially available wound dressings in terms of Tegaderm and tissue adhesive products. Through cell studies and animal models of rat skin injuries and skin injuries in Bama pigs, it confirmed that the composite material SIS/PAA/LAP demonstrated excellent cell compatibility (~ 95% to L929 fibroblasts) and angiogenic activity. In animal experiments, the SIS/PAA/LAP wound dressing was applied to the wound site, facilitating remarkable regeneration of skin tissue at the wound by mechanisms such as the release of Mg^2+^ and Si^4+^ (Scheme 1). This material holds great promise for applications in the fields of wound dressings, hernia repair patches, and cardiovascular stents.

## Materials and methods

### Materials

Acrylate acid was purchased from Aladdin (China), LAP was purchased from Macklin (China), methacrylated gelatin (Gel-MA) was obtained from Sigma (50% degree of methacrylation), SIS patches were provided from Beijing Biosis Healing Biological Technology Co., Ltd. Other reagents except otherwise statement were of analytical grade and supplied by Beijing Chemical Reagent Co., Ltd. (China).

### LAP gelling study

To disperse LAP nanoparticles at different concentrations in deionized water (2 wt.%, 4 wt.%, 6 wt.%, 8 wt.%, and 10 wt.%), ultrasonic dispersion was employed for 5 min at 300 W. Subsequently, the gelation time was determined by using an inverted centrifuge tube with a timing method.

### Preparation on the composite wound dressing

Different concentrations of AA (10 wt.%, 20 wt.%, 30 wt.%, and 40 wt.%) and 100 mg of Gel-MA were dissolved in 10 mL of deionized water. Additionally, 0.2 wt.% of α-ketoglutaric acid was added as a photo-initiator. The AA polymerization and photo cross-linking was initiated under UV light (300 W) for 30 min. Note that the weight percentage of AA was compared to the water weight.

Likewise, for the SIS patch, the AA pre-polymerized solutions and various concentrations of LAP nanoparticles were coated on its surface. Polymerization was initiated under UV light to fabricate SIS/PAA and SIS/PAA/LAP composite materials.

### Mechanical study

The patches were cut into regular pieces (3 cm in length, 1 cm in width) and then loaded on the universal testing machine (Instron 5848, China). The tests were run at room temperature with 10 mm min^− 1^ tensile speed to measure the stress-strain curves, and the ultimate tensile strength (UTS) was corresponding to the tension maximum at elongation at break. The Young’s modulus of the wound dressing was determined by the slope of the stress-strain curve in the elastic deformation region.

### Tissue adhesion study

Shear strength test was according to ASTM F2255. The porcine skins were cut into 4 cm × 2.5 cm, then the materials were placed on one of the porcine skins covering 2 cm × 2 cm, and then the two pieces were adhered in the opposite direction at room temperature. One hour later, the universal testing machine was used to test the force-displacement curve in the shear direction at a speed of 5 mm min^− 1^. Shear strength (Pa) with porcine skins was calculated as: F_max_ (N) / area (m^2^).

### Burst strength study

Burst strength test was according to ASTM F2392. The 3 cm porcine colon was created a 5 mm defect using biopsy, then the materials (2 cm × 2 cm) were placed to cover the defect for 15 min at room temperature. The pressure pump was exerted pressure at 2 mL min^− 1^ to record the burst strength till the leakage happened.

### Cytocompatible study

Following the ISO 10993 biological testing standards, the SIS-based wound dressings were immersed in cell culture medium at a ratio of 6 cm² mL^− 1^. After 24 hours of immersion, the obtained extract was co-cultured with L929 fibroblasts (1,000 cells per well in a 48-well plate). The co-culture was maintained for 1, 2, and 3 days. Cell viability was assessed using the CCK-8 reagent at 450 nm in a microplate reader (Bio-Rad 680, USA), and live/dead cells were visualized under a confocal laser scanning microscope (CLSM, TCS SP8, Leica, Germany) after staining with calcein-AM/PI fluorescent dye (Aladdin, China).

### Angiogenic study

To assess the angiogenic potential of the extract, human umbilical vein endothelial cells (HUVECs) were used. A density of 5,000 HUVECs were seeded in a 48-well plate and incubated with the extract for 7 days. The medium was refreshed every other day. After incubation, the cells were stained for primary VEGF antibody (ab32152, Abcam, UK), FITC-labelled F-actin and the fluorescent secondary antibody, followed by incubation in the dark for 30 min. VEGF expression within the cells was observed under CLSM. The fluorescence intensity was semi-quantitatively analyzed using Image Pro Plus software.

Matrigel (Corning, USA) was used to prepare a substrate for cell seeding in cell culture plates. A density of 5,000 HUVECs were seeded and incubated with the extract for 12 hours. Tubular network formation was observed under an optical microscope. The spacing between the networks was semi-quantitatively measured using Image J software.

### Full-thickness wound model on the rat

All the animal experiments were complied with the guidelines of the Tianjin Medical Experimental Animal Care, and animal protocols were approved by the Institutional Animal Care and Use Committee of Yi Shengyuan Gene Technology (Tianjin) Co., ltd (No. YSY-DWLL-2023227). Sprague-Dawley rats after being anaesthetized by isoflurane, the full thickness skin defect was created by 10 mm biopsy punching on the back of the rats, then the animal models could be divided into four groups. (1) No further treatment, leaving the wound alone (set as control groups). (2) The wound was covered by Tegaderm™. (3) The wound was covered by SIS. (4) The wound was covered by SIS/PAA/LAP. After implanting the materials, the implanted materials were fixed and covered by the sterile bandage to prevent the implant from dropping or separating from the wound. All the implanted materials were refreshed every two days, followed by the standard of care. At 3-, 6-, 9-day post-surgery, the gross appearances of the wound regeneration were estimated, among them, the harvested located tissues were fixed in 10% formalin and sectioned for H&E staining, Masson’s trichrome staining and VEGF/DAPI (ab32152, Abcam) immunohistochemical staining. The images were captured on the digital slice scanning equipment (Nanozoomer, Hamamatsu, Japan).

### Wound model on the Bama miniature pig

All the animal experiments were complied with the guidelines of the Tianjin Medical Experimental Animal Care, and animal protocols were approved by the Institutional Animal Care and Use Committee of Yi Shengyuan Gene Technology (Tianjin) Co., ltd (No. YSY-DWLL-2023228). Two pigs after being anaesthetized by isoflurane, the skin defect was created by 20 mm biopsy punching on the back of the pig, then the animal models could be divided into four groups. (1) No further treatment as control groups. (2) The wound was covered by Tegaderm™. (3) The wound was covered by SIS. (4) The wound was covered by SIS/PAA/LAP. After implanting the materials, the implanted materials were fixed and covered by the sterile bandage to prevent the implant from dropping or separating from the wound. All the implanted materials were refreshed every two days, followed by the standard of care. At 3-, 6-, 9-, and 12-day post-surgery, the gross appearances of the wound regeneration were estimated, among them, the harvested located tissues were fixed in 10% formalin and sectioned for H&E staining, Masson’s trichrome staining, and TNF-α/CD163/DAPI (MA5-44021, ThermoFisher; EPR19518, Abcam) and VEGF/α-SMA/DAPI (ab2350, Abcam; MA1-06110, ThermoFisher) immunohistochemical staining. The images were captured on the digital slice scanning equipment. For reverse transcription-polymerase chain reaction (RT-PCR), the primers were designed and listed in Table S1, the harvested granulation tissues (1 mm×1 mm×1 mm) were immersed in Trizol (1 mL, Invitrogen, USA) to extract the total RNA. Gene expressions were calculated by 2^−ΔΔCt^ method, and expressed in relation to the expression of a housekeeping GAPDH gene.

### Statistical analysis

All quantitative data was represented as mean ± standard deviation (SD). Statistical analysis was carried out using SPSS software by one-way analysis of variance (ANOVA). Differences between groups of ^*^P < 0.05 were considered statistically significant, ^**^P < 0.01 were considered highly significant. ^***^P < 0.001 were considered very highly significant.

## Results and discussion

### Preparation and characterization on the SIS/PAA/LAP wound dressing

The LAP nanoparticles owned particle size of approximately 10 nm. TEM images showed specific elements such as Mg, Si, Na, and O (Fig. S1). We firstly investigated the gelation properties of the LAP nanoparticles based on electrostatic interactions. As shown in Fig. S2, when the LAP concentrations in water were low (e.g., 2 wt.% and 4 wt.%), the electrostatic interactions between LAP nanoparticles were weak to make gel formation difficult. As the concentration increased, gel formation became evident, and the gelation time decreased with higher nanoparticle concentrations. When the LAP concentration reached 10 wt.%, the gelation time declined to 86.75 ± 6.70 s. Then, we tested the tissue shear adhesion of PAA hydrogels formed by cross-linking with different concentrations of AA, according to ASTM F2255. The results showed that with increasing AA concentration, the shear adhesion strength increased accordingly. At 40 wt.%, the adhesion reached 23.59 ± 5.59 kPa. Considering the higher adhesion strength, we chose a 40 wt.% concentration of AA for further experiments.

**Fig. 1:**
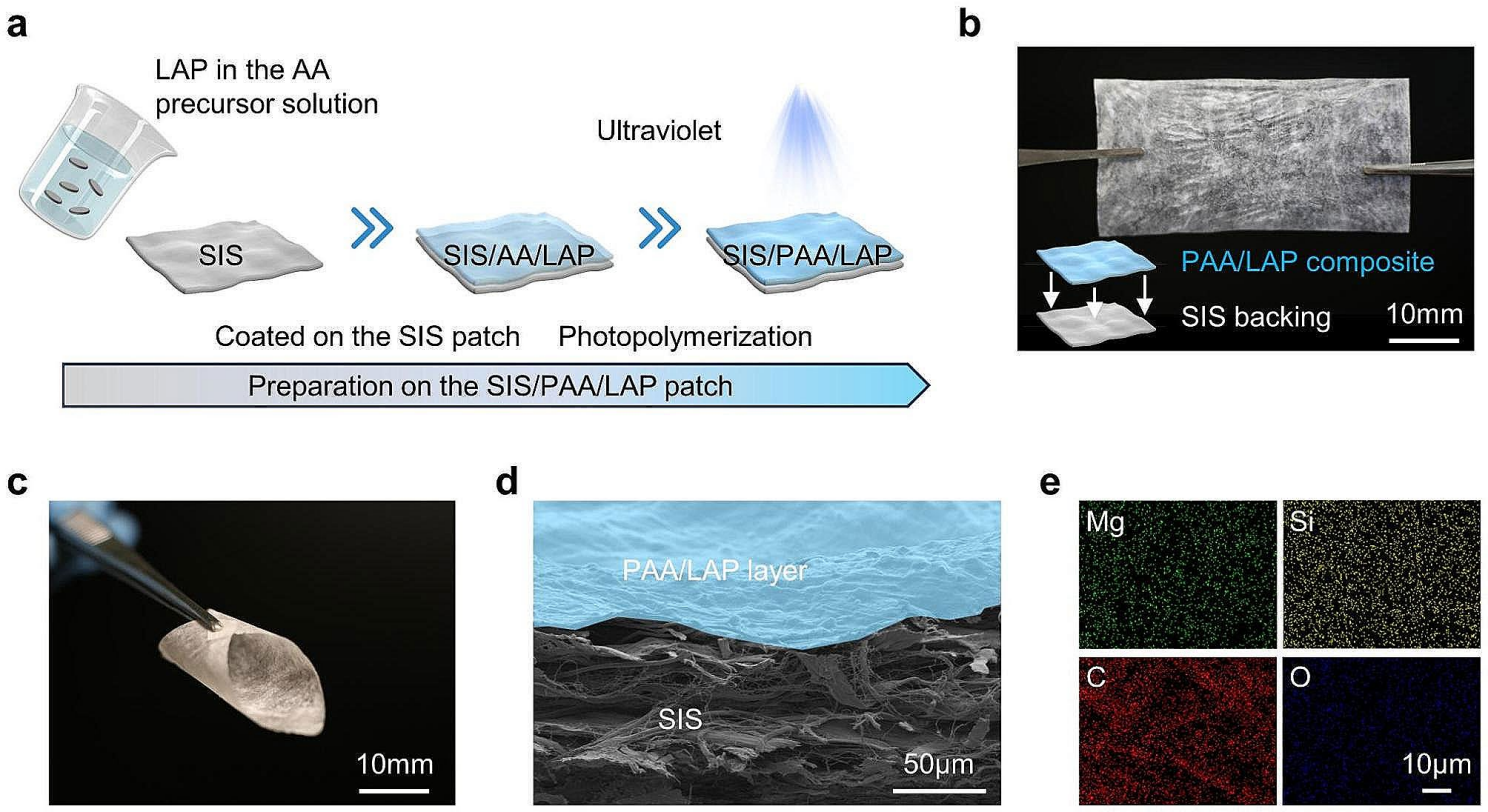
Preparation on the SIS/PAA/LAP wound dressing. **a** Schematic illustration on fabricating the SIS/PAA/LAP patch, starting from the AA pre-polymerized solution containing LAP nanoparticles coated on the SIS patch, to photopolymerization and crosslinking to form PAA/LAP adhesive layer on the SIS substrate. **b** Gross appearance on the SIS/PAA/LAP patch. **c** Flexibility of the SIS/PAA/LAP patch. **d** Cross-sectioned SEM image on the SIS/PAA/LAP patch. **e** Elemental mappings on the SIS/PAA/LAP.

Figure 1a illustrated the schematic process of preparing the asymmetric SIS-based wound dressing, which involved coating the SIS surface with AA pre-polymerized solution containing LAP nanoparticles, and then subjected it to UV light exposure, resulting in the formation of SIS/PAA/LAP patch. Figure 1b showed the physical appearance of the SIS/PAA/LAP patch, by estimating its thickness, the PAA/LAP layer owned 4.93 ± 0.67 μm, demonstrating that the PAA adhered to the SIS surface without affecting the patch’s morphology, and the SIS/PAA/LAP patch exhibited good flexibility (Fig. 1c). SEM examination of the SIS/PAA/LAP revealed that the PAA/LAP coating adhered uniformly to the SIS surface (Fig. S4, Fig. 1d). Additionally, specific element mappings of LAP, such as Mg and Si, confirmed the LAP even distribution within the SIS/PAA/LAP patch. We also conducted mechanical tests on the SIS, SIS/PAA, and SIS/PAA/LAP patches. The results confirmed that the introduction of PAA and PAA/LAP coatings improved the mechanical strength. Young’s modulus and tensile strength of SIS patches increased from 215.15 ± 13.90 MPa and 17.56 ± 3.82 MPa to 360.63 ± 24.37 MPa and 48.90 ± 6.80 MPa, respectively, in the case of SIS/PAA/LAP. This asymmetric SIS/PAA/LAP structure exhibited tissue adhesion strength, as shown in Fig. S6. These results demonstrated that through selection of LAP and AA concentrations, we were able to prepare asymmetrically adhesive SIS-based wound dressings.

### Evaluation on the tissue adhesion

**Fig. 2:**
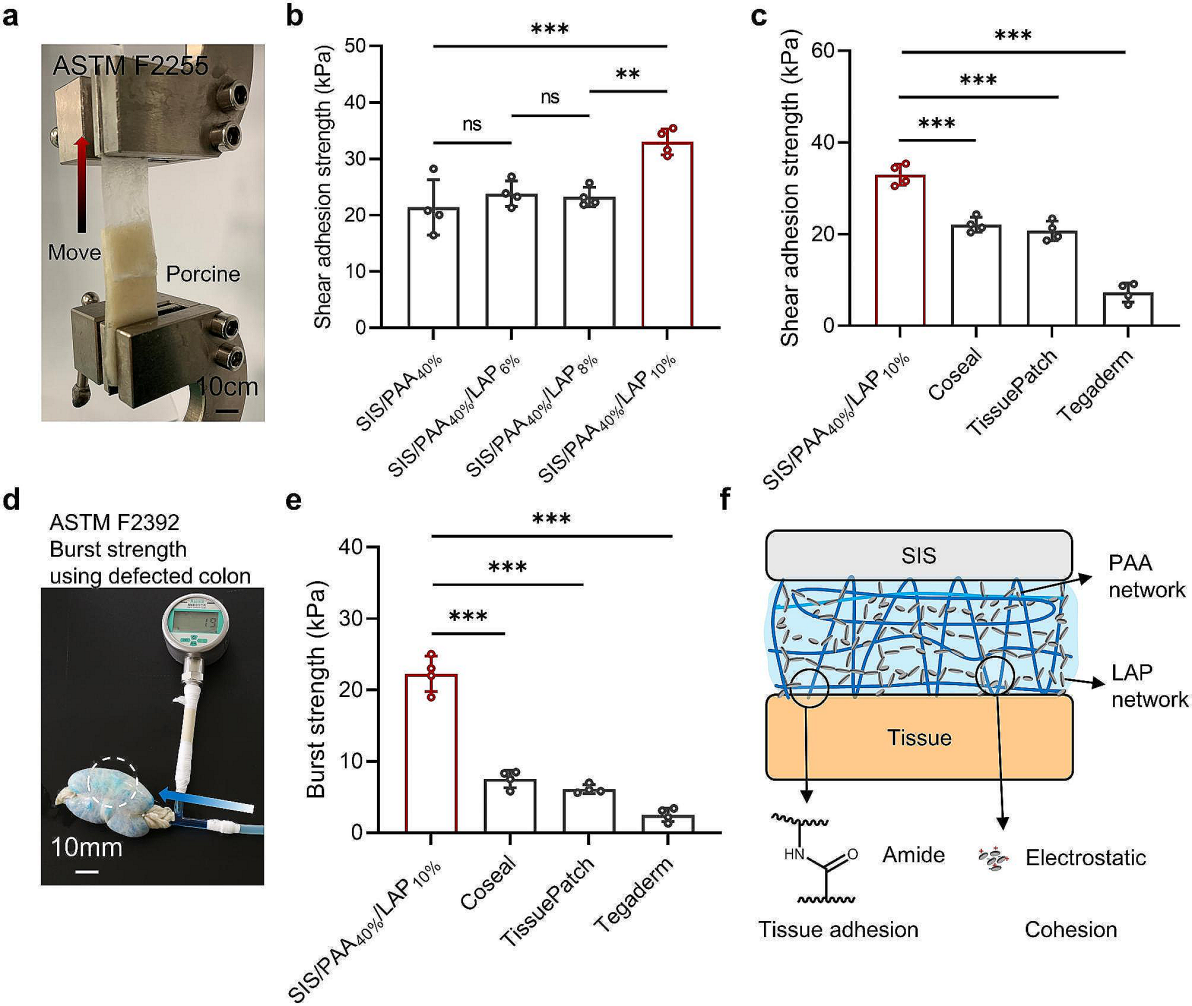
Tissue adhesion on the SIS/PAA/LAP wound dressing. **a** Lap shear adhesion assay based on ASTM F2255. **b** Shear adhesion strength with different LAP concentrations in the PAA adhesive layers. **c** Comparisons of adhesion strength among various commercial products to the SIS/PAA/LAP. **d** Burst strength assay based on ASTM F2392. **e** Comparisons of burst strength among various commercial products to the SIS/PAA/LAP. **f** Explanation on the inner double chemical-physical networks to enhance mechanical strength. *N* = 4, ^**^P ≤ 0.01, ^***^P ≤ 0.001

We conducted systematic testing to evaluate the tissue adhesion of the wound dressings. In tissue shear adhesion tests, when the LAP concentration reached 10 wt.%, there was a significant increase in shear adhesion strength, rising from an initial value of 21.38 ± 4.94 kPa (SIS/PAA_40%_) to 32.98 ± 2.31 kPa (SIS/PAA_40%_/LAP_10%_) (Fig. 2**(a, b)**), which was attributed to the formation of a second crosslinking network and cohesion beside the PAA network. Furthermore, we compared the adhesion strength of the prepared SIS/PAA/LAP with commercial tissue adhesive products, demonstrating that its adhesion strength surpassed products such as Coseal (22.05 ± 1.65 kPa), TissuePatch (20.70 ± 2.12 kPa), and Tegaderm (7.25 ± 2.08 kPa). Additionally, we tested the burst strength according to the ASTM F2392 standard. The results revealed that the SIS/PAA/LAP had burst strength of 22.25 ± 2.50 kPa, significantly higher than Coseal (7.53 ± 1.22 kPa), TissuePatch (6.10 ± 0.62 kPa), and Tegaderm (2.50 ± 0.95 kPa) (Fig. 2**(d, e)**). We assumed that the combination of double network structures including LAP electrostatic interaction as the second cohesion, and PAA chemical crosslinking hydrogel as the tissue adhesion and first cohesion layers significantly enhanced the material’s mechanical strength and adhesion strength (Fig. 2f) [20, 21]. Consequently, the prepared SIS/PAA/LAP wound dressing exhibited excellent tissue adhesion, potentially facilitating wound closure and preventing issues such as the detachment of dressings from the wound site. For ease to describe the different groups, in the following assays we remarked SIS/PAA_40%_ and SIS/PAA_40%_/LAP_10%_ as SIS/PAA and SIS/PAA/LAP to conduct assays.

### Cellular response to the SIS/PAA/LAP

**Fig. 3:**
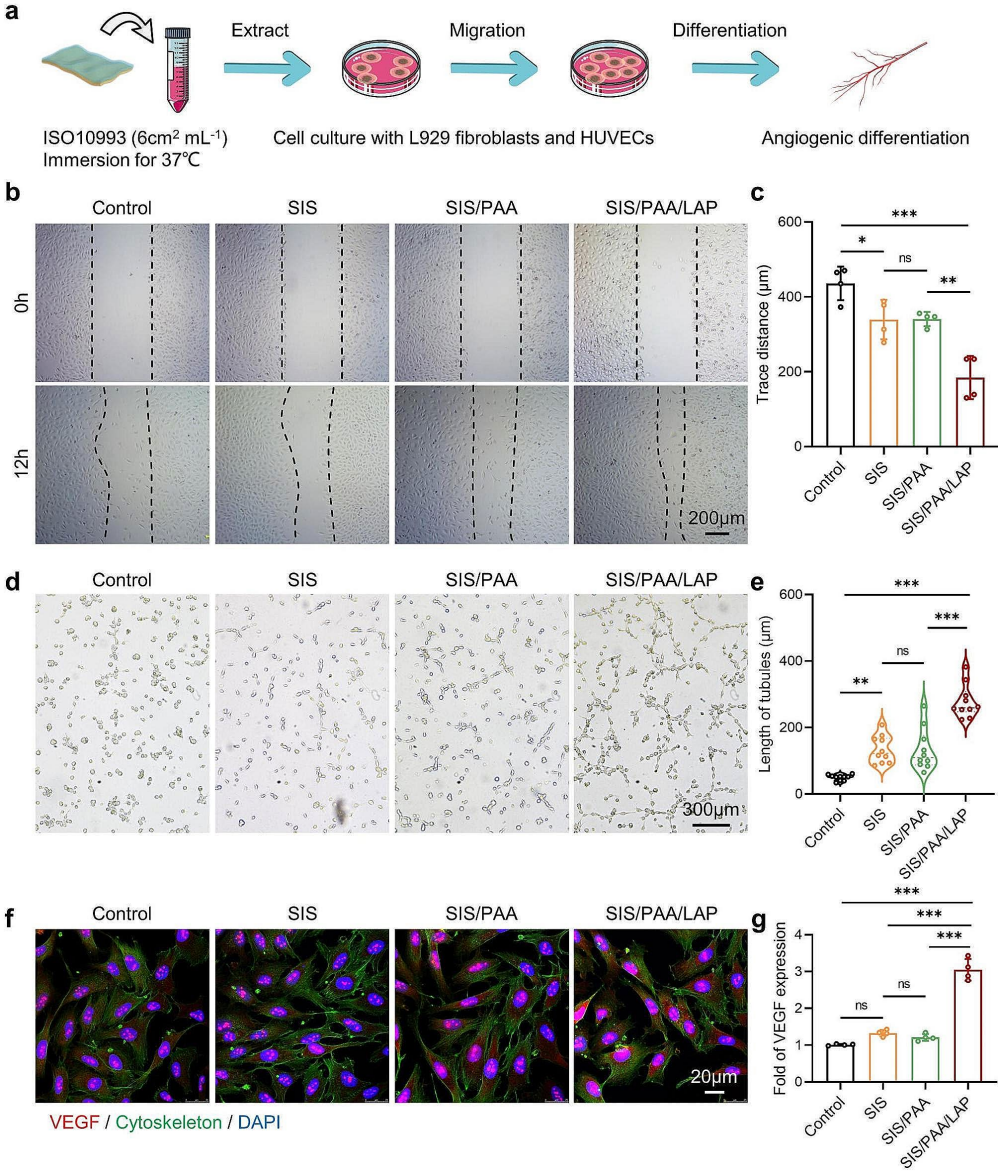
Angiogenesis assays of the SIS/PAA/LAP wound dressing in vitro. **a** Scheme on preparing the conditioned medium for inducing angiogenic differentiation. **b** HUVECs scratch assay to estimate cell migration distance. **c** Semi-quantitative analysis of trace distance based on panel **b**. **d** Tube formation assay of HUVECs after 12-hour incubation. **e** Semi-quantitative analysis of tubule length based on panel **d**. **f** Immunofluorescent staining of VEGF (red) /cytoskeleton (green) / DAPI (blue) after 7-day incubation. **g** Semi-quantitative analysis of VEGF fluorescent intensity based on panel **f**. *N* = 4, ^*^P ≤ 0.05, ^**^P ≤ 0.01, ^***^P ≤ 0.001

We first conducted cytotoxicity evaluation following ISO10993 standard by soaking the materials in cell culture media for 24 hours. The resulting extraction medium was then used to assess the cytotoxicity of the SIS/PAA/LAP on L929 cells and HUVECs (Fig. 3a). As shown in Fig. S7, the results from co-culturing with L929 cells showed that the SIS/PAA/LAP groups displayed spindle-shaped cell morphology, indicating that it did not induce significant cytotoxicity. Moreover, with extending culture period (up to 3 days), cell proliferation was observed, indicating good cell compatibility of the extraction medium. Next, we conducted cell scratch, tube formation, and angiogenesis differentiation assays on HUVECs. Figure 3**(b, c)** showed the extracts from the SIS/PAA/LAP significantly promoted HUVEC migration, with a migration rate ~ 4.86 times than that of the control. Additionally, a modest effect of the SIS in promoting cell migration was observed, possibly due to the presence of residual bioactive components such as TGF-β in the material [14].

In the tube formation experiment, the SIS/PAA/LAP showed denser tube numbers, junction numbers, and longer tube lengths (~ 278 μm), while the control owned ~ 50 μm, and the SIS had ~ 133 μm (Fig. 3**(d, e)**). In the immunofluorescence staining, the control group showed lower VEGF expressions, while in the SIS/PAA/LAP, VEGF expression was ~ 3 times than that of the control (Fig. 3**(f, g)**). These results suggested that the release of bioactive ions such as Mg^2+^ and Si^4+^ from the SIS/PAA/LAP could intensively enhance cell migration and angiogenesis, indicating its potential for effective tissue repair in the field of soft tissue regeneration.

### Tissue regeneration in the animal model

**Fig. 4:**
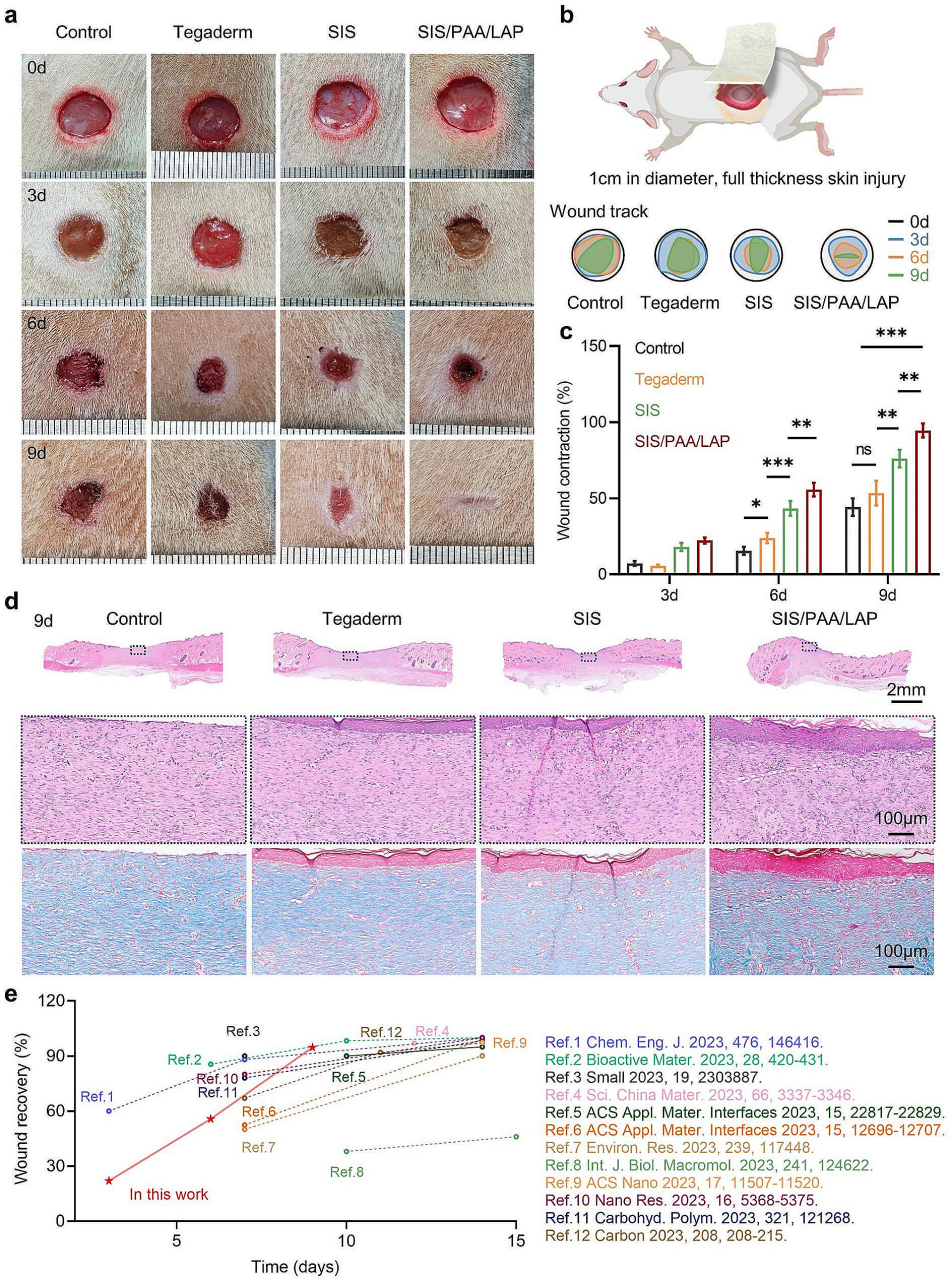
Wound regeneration in the rat full-thickness skin injury. **a** Gross appearance on the wound repair. **b** Real-time wound trajectory on the wound regeneration. **c** Wound contraction along with time. **d** H&E and Masson’s trichrome staining on the located wound tissues after 9-day. **e** Our current work compared with recent wound healing materials. *N* = 4, ^*^P ≤ 0.05, ^**^P ≤ 0.01, ^***^P ≤ 0.001

In a full-thickness skin injury model in rats, we initially established circular defects with a diameter of 10 mm on the backs of anesthetized rats. Different wound dressings (e.g., Tegaderm, SIS, and SIS/PAA/LAP) were placed on the wound surface, and wound dressings were changed every other day following standard care procedures [22]. Upon macroscopic assessment of the wound healing, the control and Tegaderm groups showed poor wound repair, with noticeable wounds after 9 days. The SIS groups had certain wound repair at day-9, while the SIS/PAA/LAP groups had mostly recovered wounds (Fig. 4a), and a similar tissue repair rate was visible in the semi-quantitative wound tracking analysis (Fig. 4b). Statistically, postoperative wound contraction at day-6 was 15.45 ± 2.60% for the control groups, 23.88 ± 3.35% for Tegaderm, 43.28 ± 4.79% for SIS, and 55.70 ± 4.42% for SIS/PAA/LAP. At day-9, the wound contraction was 44.20 ± 5.75% for the control group, 53.40 ± 8.17% for Tegaderm, 76.10 ± 5.78% for SIS, and 94.58 ± 4.54% for SIS/PAA/LAP (Fig. 4c).

Combining the former cellular results (Fig. 3), we supposed that the SIS contained endogenous growth factors and other active substances such as VEGF and TGF-β [14, 23], and the SIS/PAA/LAP enabled firmly adhere to the wound surface. Additionally, the LAP nanoparticles when infiltrated by body fluids, could release bioactive ions such as Mg^2+^ and Si^4+^, which facilitated tissue healing [24–27]. Histological staining at 6 and 9 days revealed that the control and Tegaderm groups had obvious wound defects and lower collagen expression. In contrast, the wound gaps in the SIS, and particularly the SIS/PAA/LAP groups became narrower, with higher collagen mature (Fig. 4d, Fig. S8). This asymmetric adhesive SIS/PAA/LAP wound dressing with continuous ion release showed advantages in wound healing compared with other recent reports regarding on wound healing materials (Fig. 4e). Furthermore, in the VEGF/DAPI fluorescence staining, the SIS/PAA/LAP groups exhibited higher VEGF fluorescence intensity, being 1.84 times and 2.89 times than that of the control group at 6 and 9 days, respectively (Fig. S9). These findings suggested that the prepared SIS/PAA/LAP could firmly adhere to wound surfaces and, as LAP released active ions, to achieve excellent tissue repair results.

In pathological H&E staining of major metabolic organs such as the heart, liver, spleen, lung, and kidney at 9 days post-surgery, the tissue morphology and hematological analysis of the SIS/PAA/LAP groups showed no significant difference compared to the control groups, indicating good tissue compatibility (Fig. S10).

**Fig. 5:**
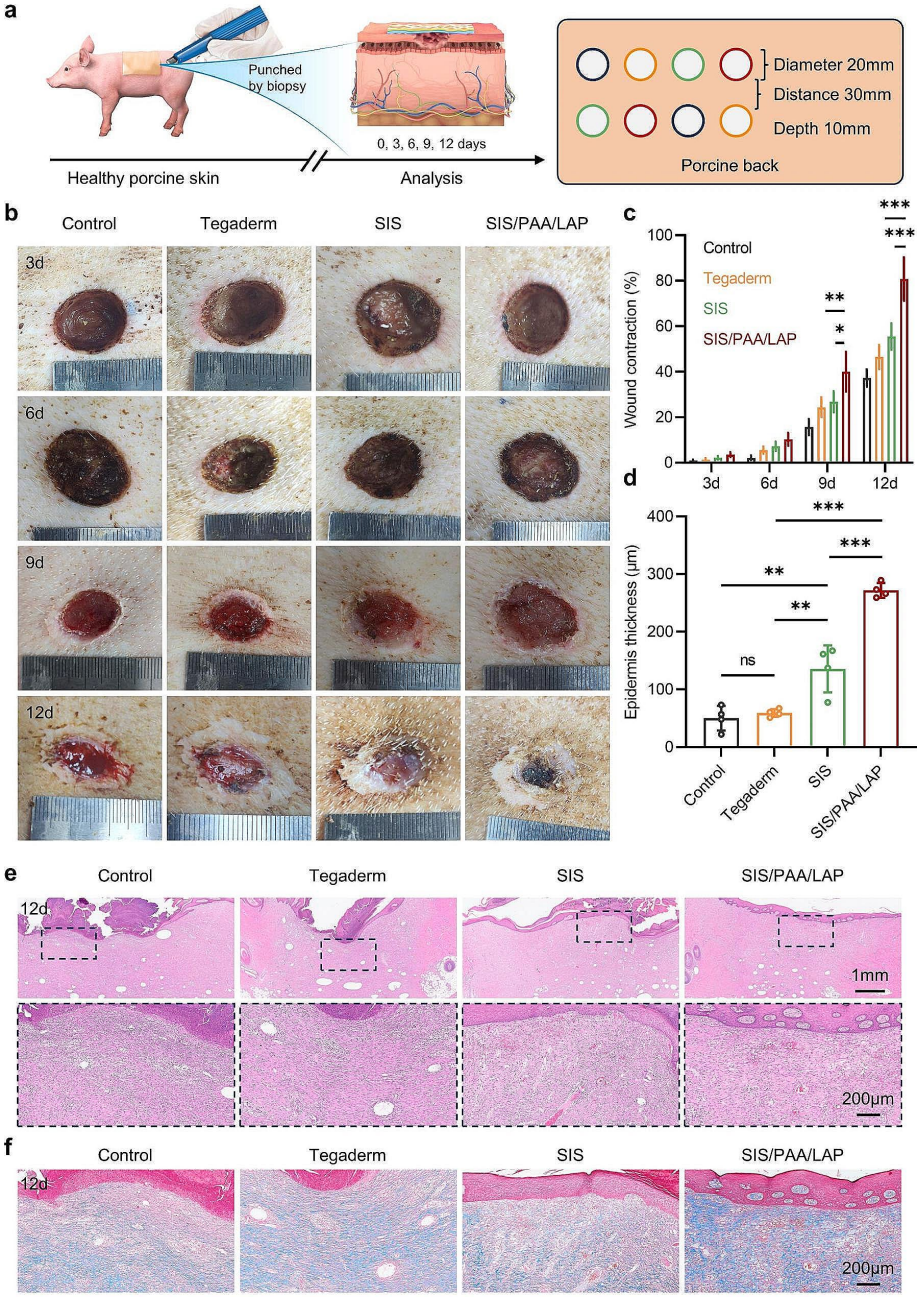
Wound regeneration in the Bama miniature porcine skin injury. **a** Illustration on the experiment routine and pathological analysis. **b** Gross appearance on the wound repair. **c** Wound contraction along with time. **d** Epidermis thickness growth after 12-day. **e** H&E staining, and **f** Masson’s trichrome staining on the located wound tissues after 12-day. *N* = 4, ^*^P ≤ 0.05, ^**^P ≤ 0.01, ^***^P ≤ 0.001

Furthermore, in a larger-scale wound defect model on the skin of miniature pig, we evaluated the effectiveness of the wound dressings in promoting tissue healing (Fig. 5a). In the overall wound repair outcome, all groups exhibited slow rate wound repair in the early stage, however, after 9 days, the wound repair results became different. At 9 days post-surgery, the SIS/PAA/LAP groups had a wound contraction rate of 40.02 ± 8.76%, significantly higher than the other groups. At day-12, the wound contraction rate of the SIS/PAA/LAP groups reached 80.75 ± 9.53%, while the control, Tegaderm, and SIS had rates of only 37.25 ± 3.77%, 46.50 ± 5.26%, and 55.50 ± 5.80%, respectively (Fig. 5**(b, c)**). In histological staining, we observed that the SIS/PAA/LAP wound dressing used on the wound surface did not induce adverse reactions. Additionally, the use of SIS and SIS/PAA/LAP led to a significant increase in epidermis thickness (SIS: 135.63 ± 40.86 μm, SIS/PAA/LAP: 271.75 ± 12.84 μm) compared to the control and Tegaderm groups. In Masson’s trichrome staining, the SIS/PAA/LAP groups exhibited higher levels of collagen expression, with more orderly collagen arrangement (Fig. 5**(d-f)**). These results confirmed that the SIS/PAA/LAP had good biocompatibility and was not only effective in promoting full-thickness skin wound repair in rats but also achieved excellent tissue repair results in a larger-scale miniature pig skin injury model.

**Fig. 6:**
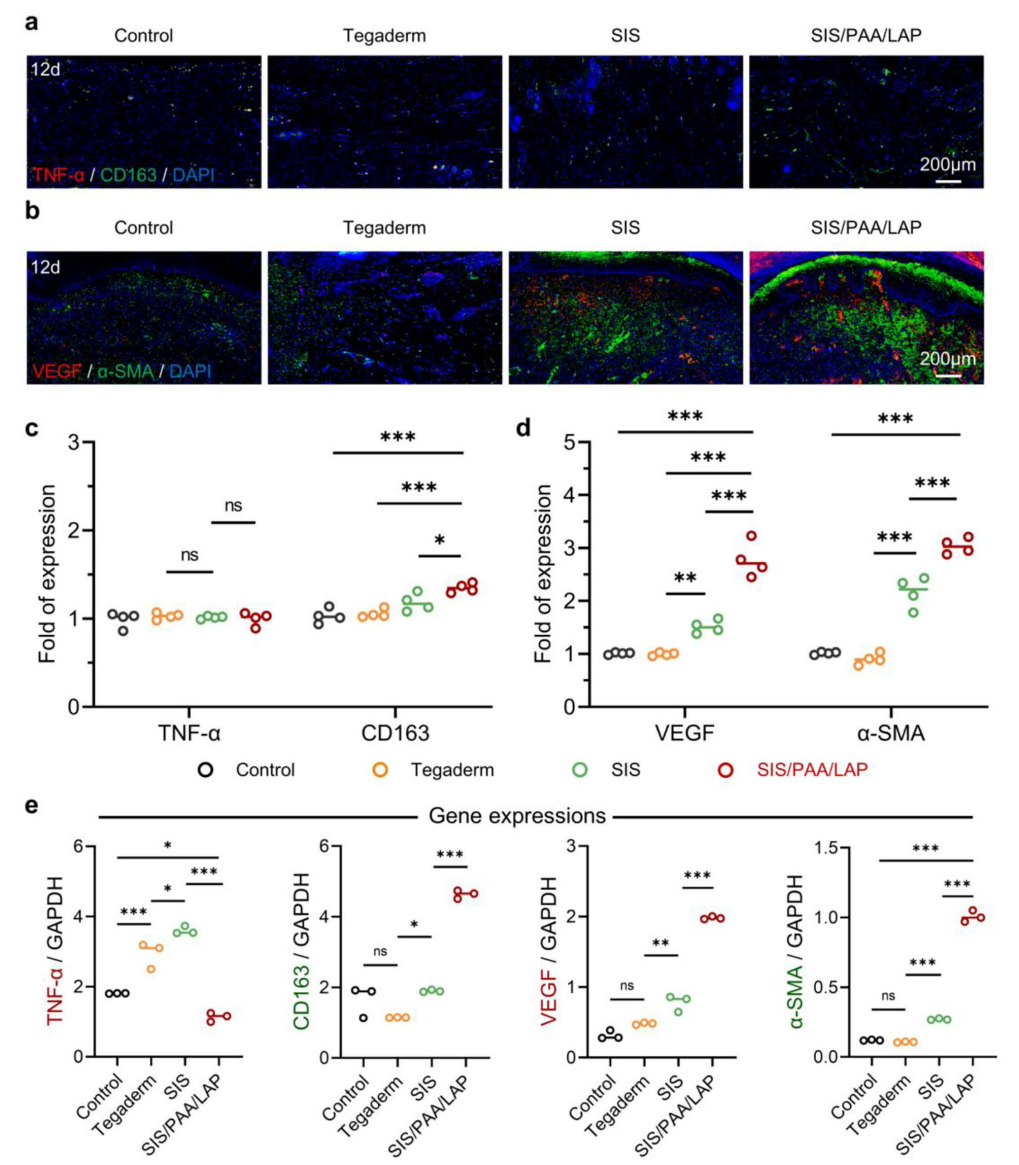
Histological analysis on the wound regeneration in pig. **a** Immunofluorescent staining of TNF-α (red) / CD163 (green) / DAPI (blue), and **b** VEGF (red) / α-SMA (green) / DAPI (blue) on the located wound tissue after 12-day. **c** Semi-quantitative analysis of TNF-α / CD163, and **d** VEGF / α-SMA fluorescent intensity expression based on panel **a** and **b**, *N* = 4. **e** RT-PCR analysis of the retrieved granulation tissues for specific gene expressions. *N* = 3, ^*^P ≤ 0.05, ^**^P ≤ 0.01, ^***^P ≤ 0.001

In addition, we conducted immunohistochemical staining on the 12-day wound tissue to further analyze how SIS/PAA/LAP achieved improved tissue repair. We chose TNF-α as a typical pro-inflammatory marker and CD163 as a typical anti-inflammatory marker. Based on the expression of TNF-α/CD163 immunofluorescence, there were no significant differences in TNF-α expression among the groups. However, the SIS/PAA/LAP groups had 1.31 times the intensity of CD163 expression compared to the control, 1.28 times compared to Tegaderm, and 1.14 times compared to SIS (Fig. 6**(a, c)**). In terms of VEGF/α-SMA expression, the SIS/PAA/LAP groups exhibited higher expression levels of VEGF and α-SMA compared to the other groups. The VEGF was 2.75 times than that of the control, 2.79 times than that of Tegaderm, while α-SMA was 2.99 times than that of the control, 3.36 times than that of Tegaderm, and 1.41 times than that of SIS (Fig. 6**(b, d)**). And from the RT-PCR analysis, the specific gene expressions (e.g., TNF-α, CD163, VEGF) were also accorded with the previous findings (Fig. 6e). These results indicated that a significant inflammatory response in the early stages of wound healing [28, 29], when SIS/PAA/LAP was used as a wound dressing, the release of bioactive ions such as Mg^2+^ and Si^4+^ enabled to increase the expression levels of intercellular anti-inflammatory factors (e.g., CD163), to create a microenvironment conducive to cell migration and proliferation [30, 31]. Additionally, Mg^2+^ and Si^4+^ could significantly promote angiogenesis in tissues [27, 32, 33]. Therefore, SIS/PAA/LAP was capable of achieving excellent tissue wound repair outcomes.

## Conclusion

In this study, the asymmetric adhesive wound dressing SIS/PAA/LAP was prepared from photopolymerizing and crosslinking AA pre-cursor solution containing LAP nanoparticles on the SIS patch. By optimizing the composition (e.g., 40 wt.% AA and 10 wt.% LAP), the resulting wound dressing exhibited excellent tissue adhesion strength (~ 33 kPa) and burst strength (~ 22 kPa), which outperformed commercially available tissue adhesives used as wound dressings. In the cell assays, rat and porcine skin injury models, the SIS/PAA/LAP demonstrated good cytocompatibility and pro-angiogenic capacity, leading to remarkable tissue repair outcomes. This SIS/PAA/LAP patch not only showed advantages as wound dressings, but also held promise for applications in surgical fields, such as general and cardiac surgery.

### Electronic supplementary material

Below is the link to the electronic supplementary material.

Supplementary Material 1

## Data Availability

Data will be made available on request.
