# Supplementary material for: Asymmetric adhesive SIS-based wound dressings for therapeutically targeting wound repair
Source: J Nanobiotechnology. 2024 Jan 19;22:34. doi: 10.1186/s12951-024-02294-x (PMC10797997; doi:10.1186/s12951-024-02294-x)
Supplement: Supplementary file 1 — Supplementary Material 1 [file 12951_2024_2294_MOESM1_ESM.docx]

**Supporting Information**

**Asymmetric adhesive SIS-based wound dressings for therapeutically targeting wound repair**

Wende Yao^1,2,#^, Zelong Song^1,3,#^, Xiaodong Ma^4,#^, Yiqian Huang^5^, Xueying Zhang^5^, Yunhuan Li^5^, Pengfei Wei^5^, Julei Zhang^2,6^, Chenlu Xiong^1,2^, Sihan Yang^1,2^, Yujian Xu^2^, Wei Jing^5^, Bo Zhao^5,*^, Xuesong Zhang^1,3,*^, Yan Han^1,2,*^

^1^ School of Medicine, Nankai University, Tianjin,300071, China

^2^ Department of Plastic and Reconstructive Surgery, The First Medical Centre, Chinese PLA General Hospital, Beijing, 100853, China

^3^ Department of Orthopaedics, The Fourth Medical Centre, Chinese PLA General Hospital, Beijing, 100048, China

^4^ Department of Neurosurgery, The First Medical Centre, Chinese PLA General Hospital, Beijing, 100048, China

^5^ Beijing Biosis Healing Biological Technology Co., Ltd, Beijing 102600, China

^6^ Department of Burn and Plastic Surgery, The 980st Hospital of the PLA Joint Logistics Support Force, Hebei, China

#These authors contribute equally to this work.

*Corresponding authors.

E-mail: zhaobo@biosishealing.com (Prof. Bo Zhao), zhangxuesong301@126.com (Prof. Xuesong Zhang), and 13720086335@163.com (Prof. Yan Han).


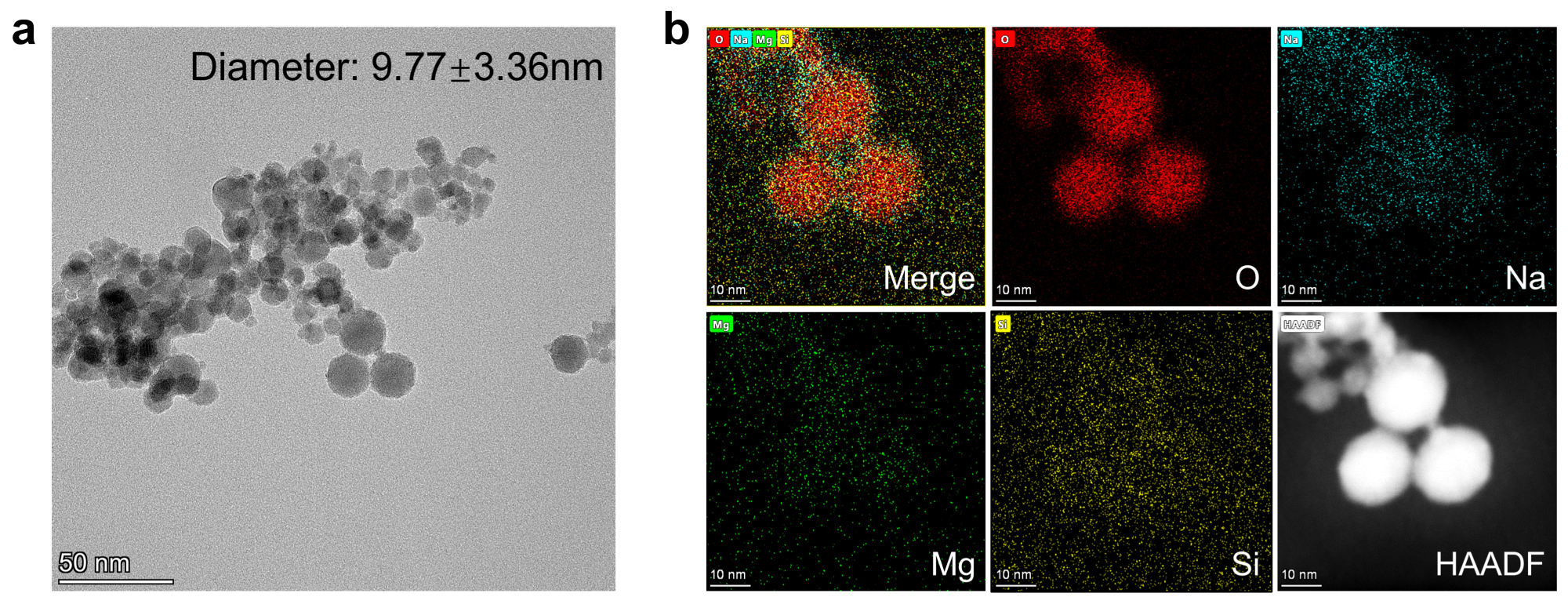


**Fig. S1** Characterization on the LAP nanoparticles. **a** TEM image. **b** Elemental mappings and HAADF images


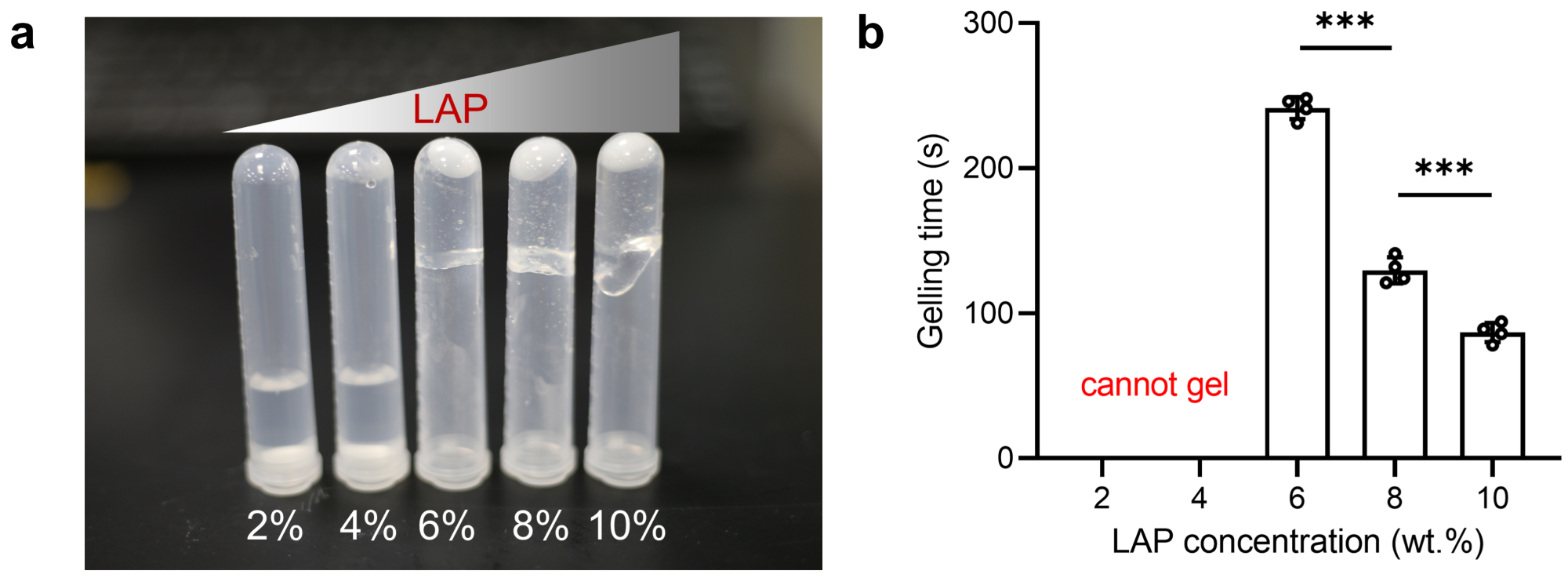


**Fig. S2** Gel formation for the LAP nanoparticles dispersed in water. **a** Gross appearance on the gelling process with different concentrations of LAP. **b** Gelling time for different concentrations of LAP.


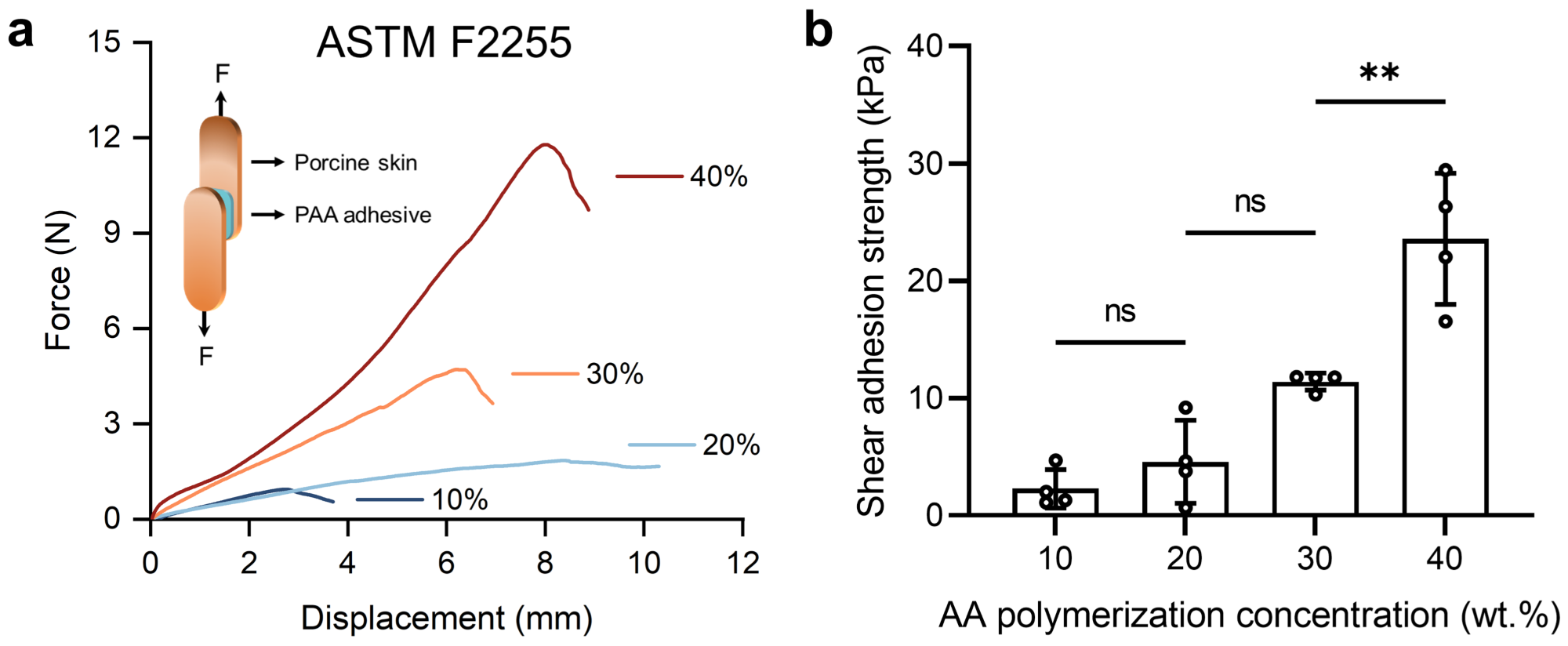


**Fig. S3** Tissue adhesion on the PAA adhesives. **a** Lap shear force-displacement for the different concentrations of AA-polymerized PAA adhesives. **b** Lap shear adhesion strength of different AA concentrations of PAA adhesives. N = 4, ^**^P ≤ 0.01.


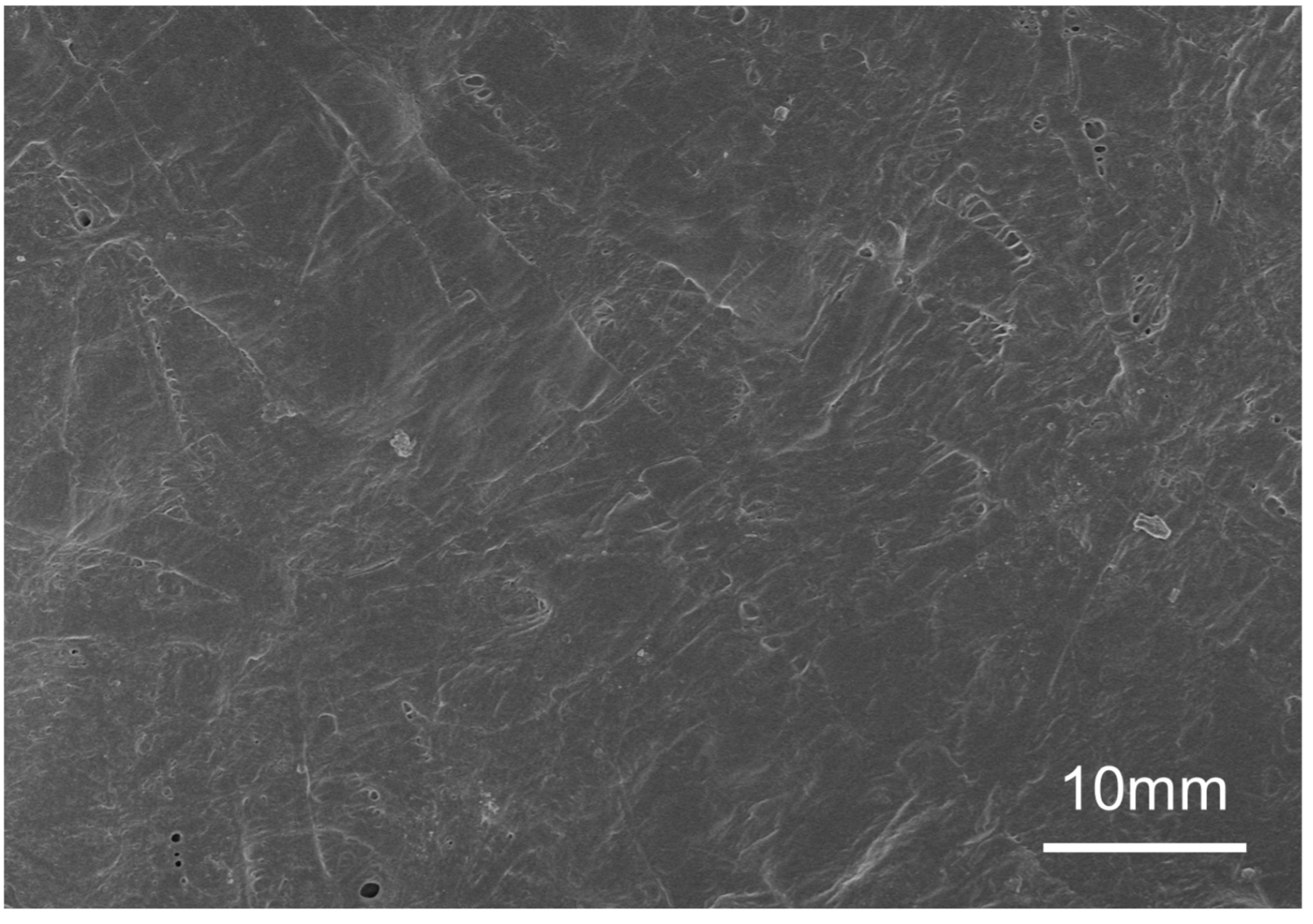


**Fig. S4** Surface morphology on the SIS/PAA/LAP wound dressings, the SEM showed the PAA/LAP side.


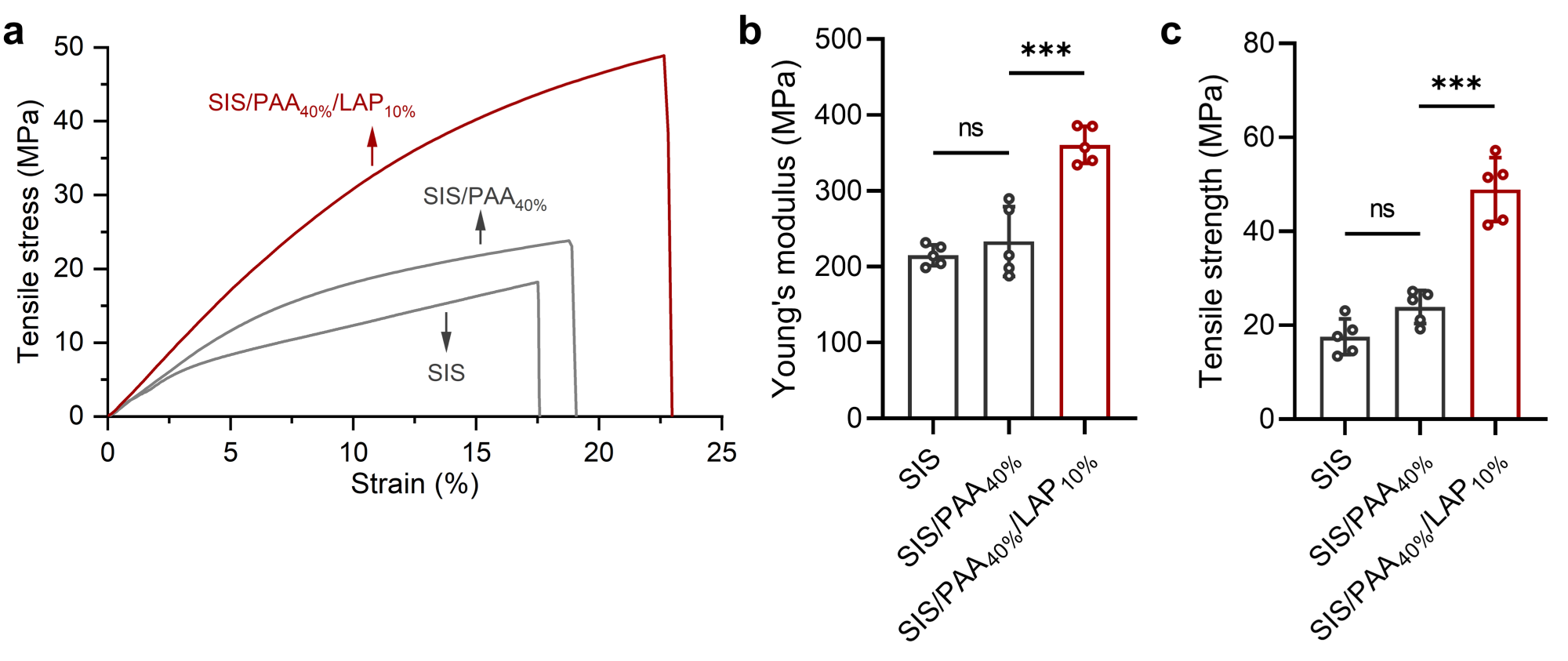


**Fig. S5** Mechanical evaluation on the SIS/PAA/LAP wound dressings. **a** Tensile stress-strain curves for the SIS, SIS/PAA_40%_, SIS/PAA_40%_/LAP_10%_ wound dressings. **b** Young’s modulus. **c** Tensile strength. N = 5, ^***^P ≤ 0.001.


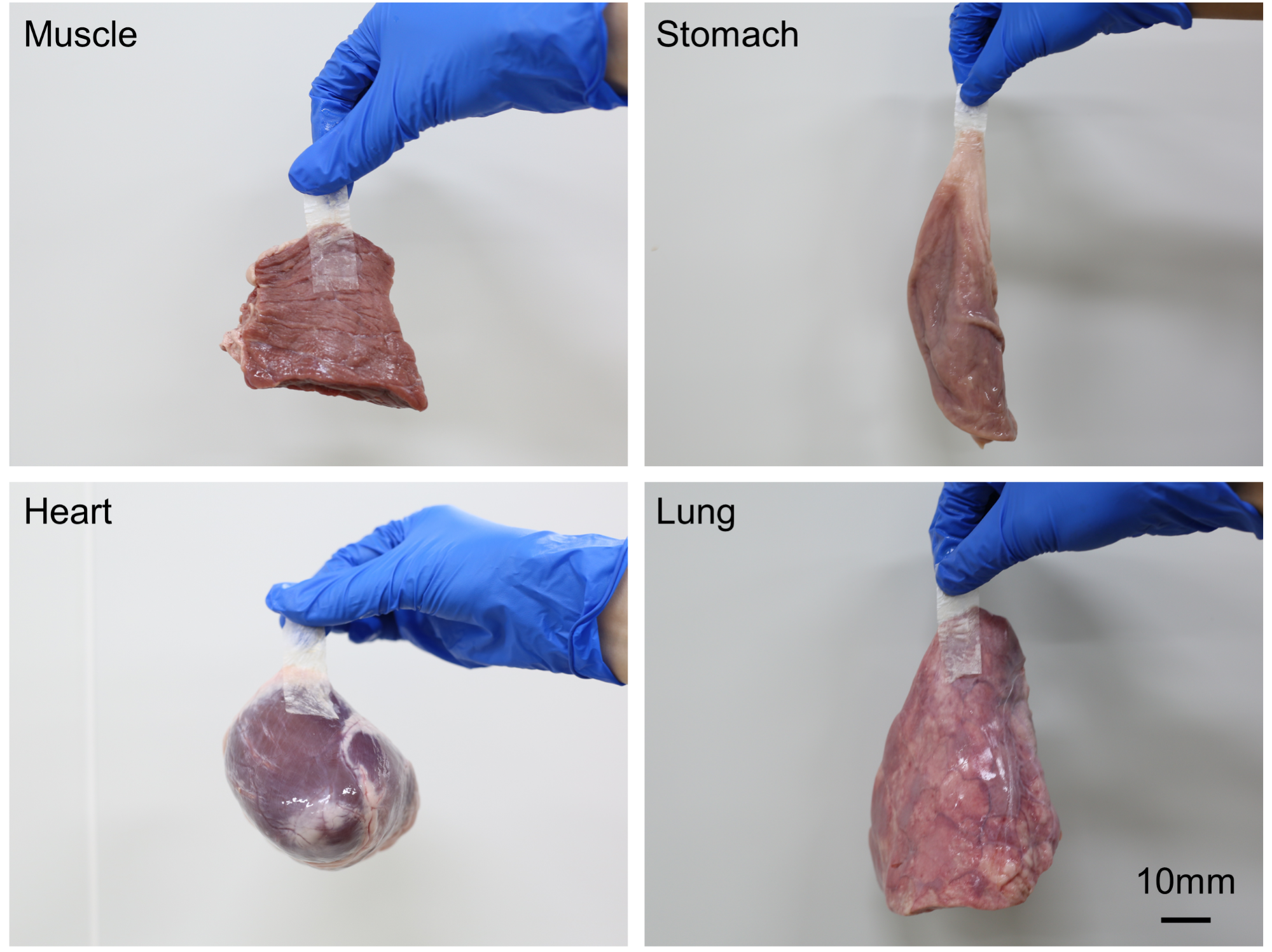


**Fig. S6** Various adhesion including muscle, stomach, heart, lung of the SIS/PAA/LAP.


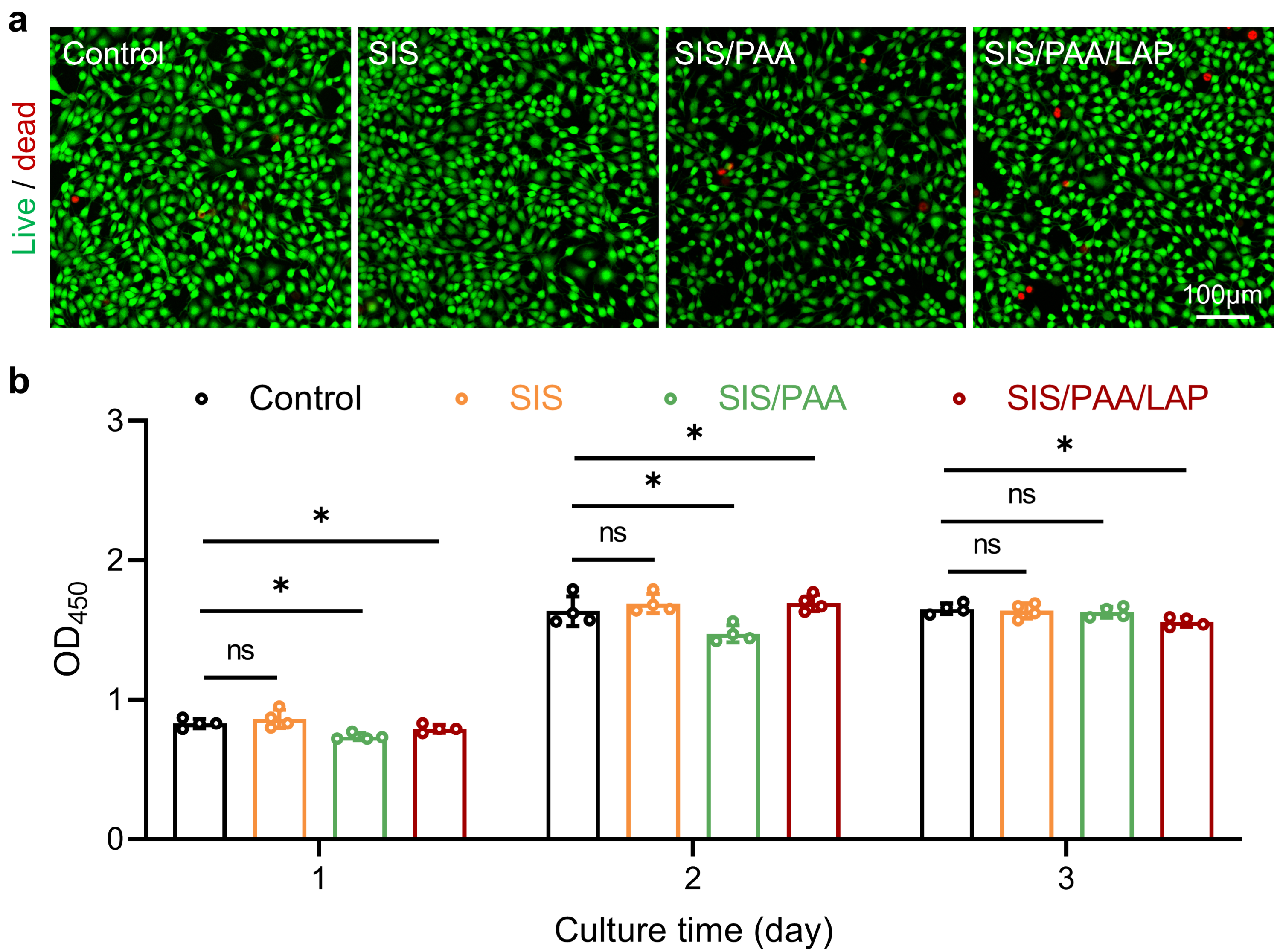


**Fig. S7** Cytocompatibility with L929 fibroblasts. **a** Live/dead staining using the conditioned extracts with L929 cells for one day. **b** Cell proliferation with extended culture time (1-3 days) to show the cell growth. N = 4, ^*^P ≤ 0.05.


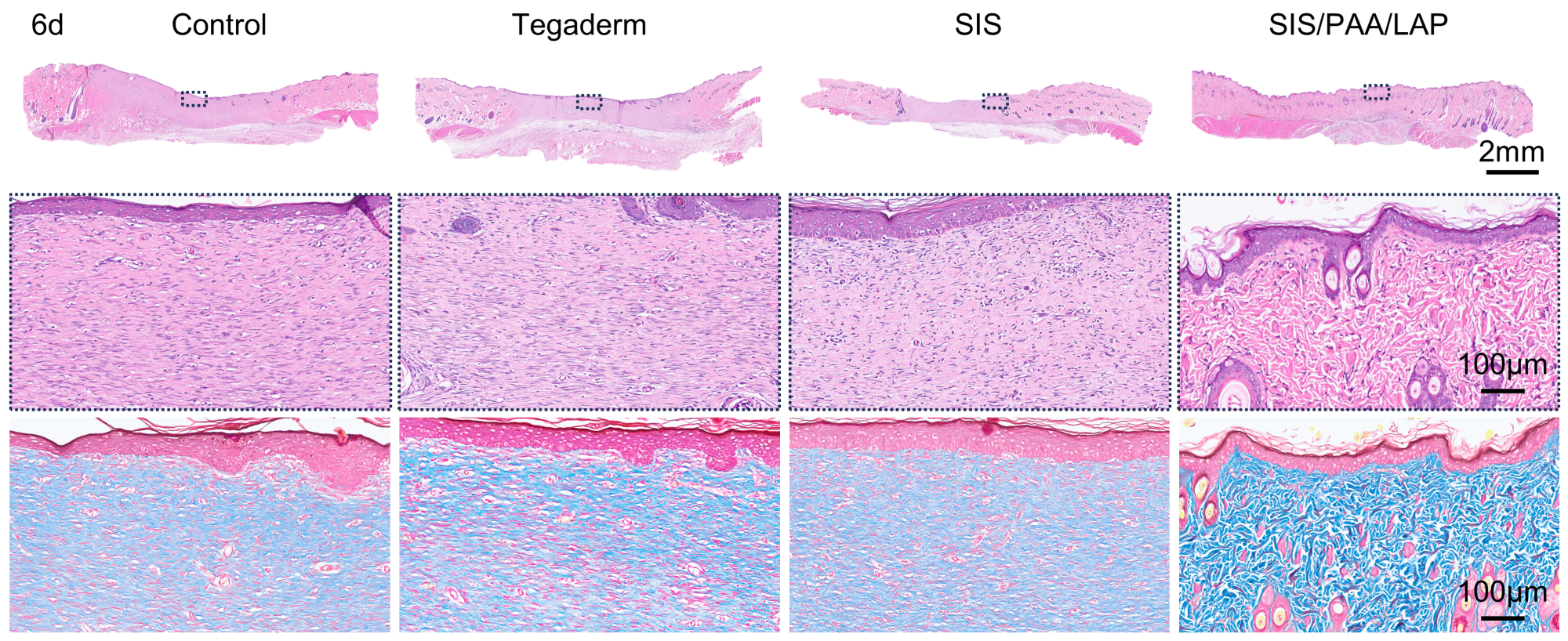


**Fig. S8** H&E and Masson’s trichrome staining on the located wound tissues after 9-day.


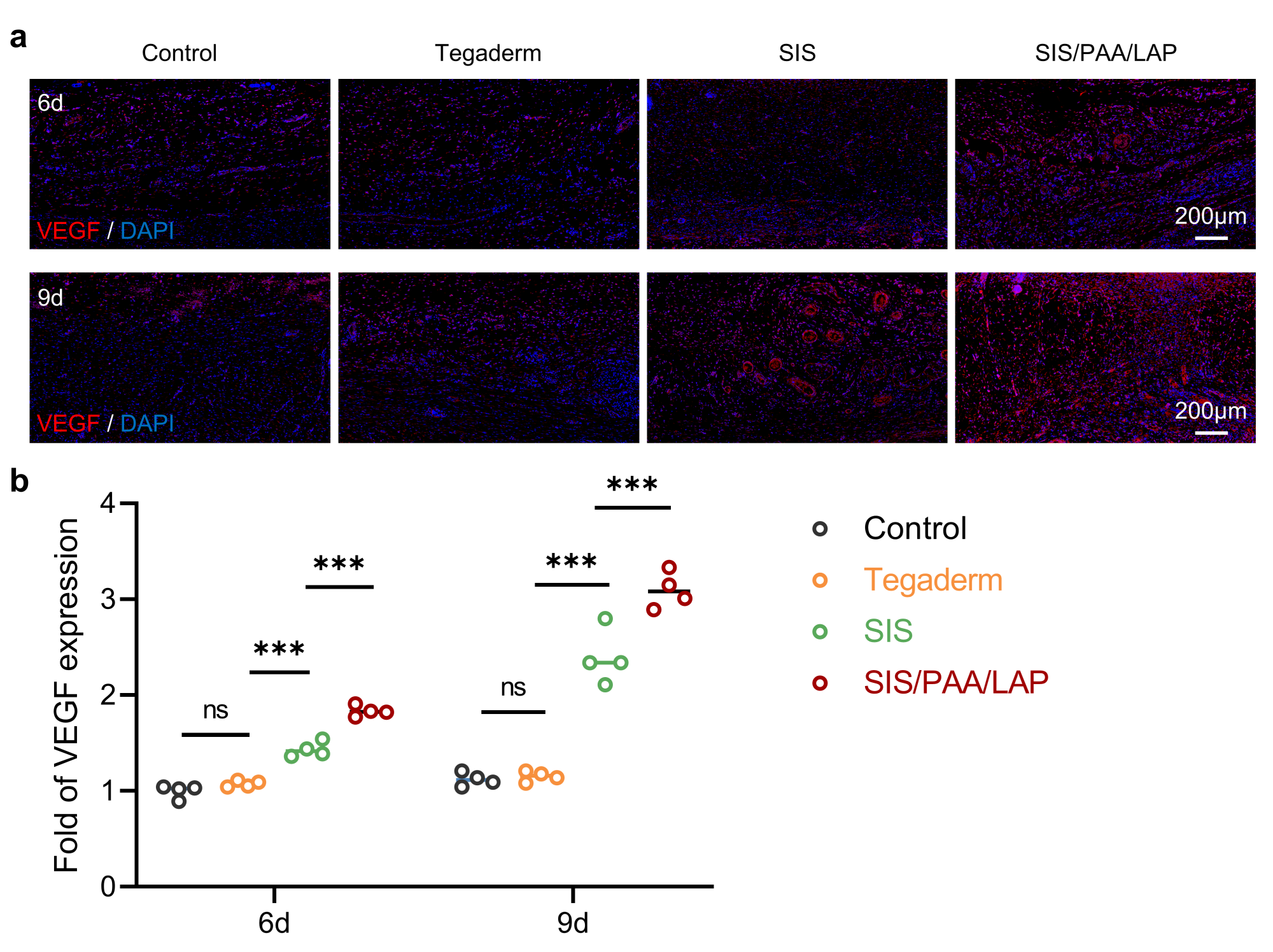


**Fig. S9** Vascularization on the wound regeneration in the rat full-thickness wound model. **a** Immunofluorescent staining of VEGF (red) / DAPI (blue) after surgery for 6- and 9-day. **b** Semi-quantitative analysis of VEGF fluorescent intensity expression based on panel (a). N = 4, ^***^P ≤ 0.001.


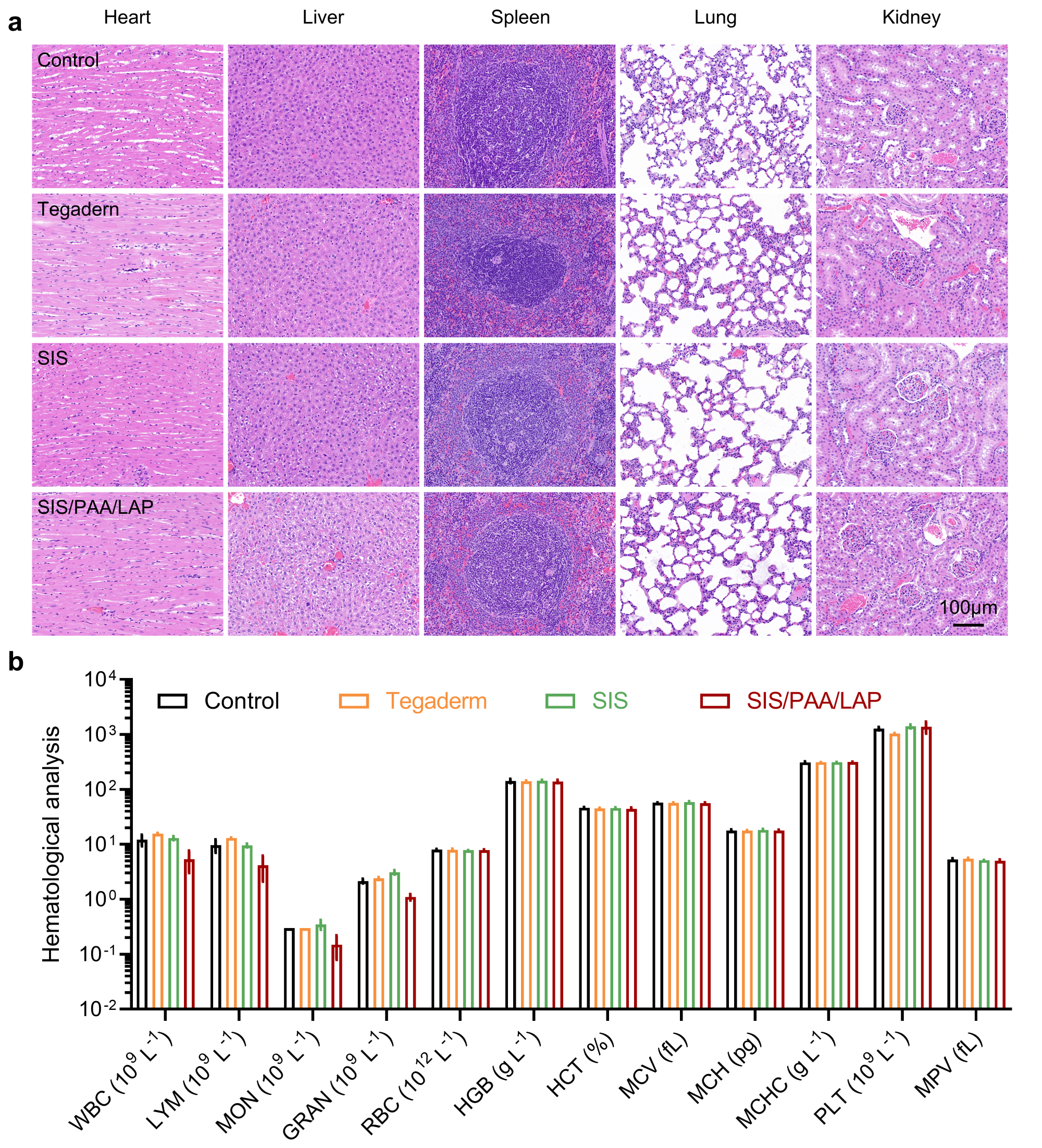


**Fig. S10** Evaluation on the biocompatibility. **a** Major organs including heart, liver, spleen, lung and kidney when the wound dressings had been implanted to the wound after 9-day post-surgery. **b** Hematological analysis after 9-day.

**Table S1.** Specific primers designed for determining gene expressions via RT-PCR.

| **Gene** | **Forward primer** | **Reverse primer** |
| --- | --- | --- |
| TNF-α | TCTTCTGCCTACTGCACTTCG | CTCGGCTTTGACATTGGCTACA |
| CD163 | AGCGGCTCTCAGAAGAATGC | CTCAAACGCAACAGGATGCC |
| VEGF | GACGAAGGTCTGGAGTGTGT | GGCCCACAGGGATTTTCTT |
| α-SMA | CCAGAGCAATCAGGGACC | CAATGGACGGGAAAACAGCC |
| GAPDH | GTCGGAGTGAACGGATTTGGC | CTTGCCGTGGGTGGAATCAT |
